# Medical Fashions

**Published:** 1882-12

**Authors:** 


### Editorial.

### MEDICAL FASHIONS.

The medical world, like the social world, is swayed by fashion. Not so completely perhaps, but as surely. Indeed there seems to be in mankind, if not a natural tendency, at least, an early acquired habit of observing times and seasons in the ordinary pursuits of life. In the days of childhood, toys and sports have their own allotted time, and the youngster, scarcely able to toddle alone, knows his "top-time," "kite-time," "marble-time," etc.; and he is still submissively obedient to the edict of fashion in the selection of his more ambitious and more active amusements. So it is when we have put away childish things, and in the real or fancied plenitude of our power become men—robust, vigorous men. Even then habit asserts itself, and holds us in subjection when the toys and sports of infancy give place to the instruments and vocations of manhood; we are still, we say, under the domination of our second, if not of our first nature, and the habit of following the fashion enfolds us in its irresistible embrace.

The admitted fact that there is an observance of fashion in medicine is not to be unconditionally decried. It is not so harmful as at first it may appear. Nay, it can be shown, we think, that like fashion in clothes, it has its advantages and that it serves indirectly to promote medical interests. For instance, it insures a certain amount of uniformity in study, observation and investigation. It serves to concentrate and fix the attention simultaneously of large numbers of workers, and to combine, as it were, the energies of the industrious members of the profession.

A master, or it may be only an enthusiast, leads the way into the misty realms of the unknown, or into a region enshrouded in doubt, and forthwith an army of eager recruits is found to follow, who, with varying degrees of ability and endowed with a full assortment of mental gifts, apply the tests of experience to the novel theory, suggestions or procedure, and so, by a multitude of witnesses the truth is established.

Of course, there is abundant room here for mistakes, and the uneducated or over-credulous may easily be led astray; but we have great confidence in the corrective influence of numerous exponents and of time, and those medical practitioners who are so completely captivated by the new and who are so easily influenced by example, like the fop and the devotee of social fashions, are usually well-meaning and amiable fellows whose opportunities for inflicting damage is happily circumscribed.

